# *Acanthamoeba* keratitis related to contact lens use in a tertiary hospital in China

**DOI:** 10.1186/s12886-019-1210-2

**Published:** 2019-09-18

**Authors:** Weiwei Li, Zhiqun Wang, Jinghao Qu, Yang Zhang, Xuguang Sun

**Affiliations:** 1 Tianjin Eye Hospital, Tianjin Ophthalmology and Visual Development Key Laboratory, Clinical College of Ophthalmology, Tianjin Medical University, Address: 4, Gansu Road, Heping District, Tianjin, 300020 China; 2 Beijing Institute of Ophthalmology, Beijing Tongren Eye Center, Beijing Tongren Hospital, Beijing Ophthalmology & Visual Sciences Key Laboratory, Capital Medical University, Address: 17 Hou Gou Lane, Chong Nei Street, Beijing, 100005 China

**Keywords:** *Acanthamoeba* keratitis, Contact lens, Genotype

## Abstract

**Background:**

To report the clinical and microbiological features of *Acanthamoeba* keratitis (AK) related to contact lens use in a tertiary hospital in China.

**Methods:**

In this retrospective study, the medical results of 61 cases of AK related to contact lens use from January 2000 to December 2017 were reviewed. The data included patients’ demographics, lens type, history, risk factors, disease stages, corneal scraping and culture reports, and treatments. Moreover, genotypic identification of some of the isolates was carried out with a PCR assay and sequence analysis of the 18S ribosomal DNA gene.

**Results:**

There were 64 eyes included in the study. A total of 32.8% of the patients wore soft contact lenses, and 67.2% of patients used overnight orthokeratology. In the cases (20 eyes) in the early stage, 65% (13 eyes) had positive results according to Giemsa-stained smears, and 0.9% sodium chloride (NaCl) wet mounts revealed trophozoites in 7 eyes (35%). Six eyes (30%) were diagnosed by confocal microscopy combined with clinical signs. In the orthokeratology patients, 87.8% (36/41) rinsed their lenses and/or cases with tap water; 55% of soft-lens wearers wore their lenses while showering. The genotype of 9 isolates was determined, and all the strains belonged to genotype T4. In the orthokeratology group, the number of patients who required therapeutic penetrating keratoplasty after 2005 was less than that before 2005 (chi-square test, *χ*^2^ = 4.209, *P* = 0.04).

**Conclusions:**

More than two-thirds of the cases were associated with orthokeratology. Examinations with Giemsa-stained smears, 0.9% NaCl wet mounts and confocal microscopy should be performed for patients who are highly suspected of having early-stage AK to help with early diagnosis. In the orthokeratology group, the rate of therapeutic keratoplasty after 2005 was less than that before 2005.

**Electronic supplementary material:**

The online version of this article (10.1186/s12886-019-1210-2) contains supplementary material, which is available to authorized users.

## Background

*Acanthamoeba* keratitis (AK) is a severe and vision-threatening infection of the cornea. In the 1980s, AK dramatically increased in conjunction with the increasing use of soft contact lenses (SCLs) [1]. AK occurred most frequently in contact lens wearers in developed countries [2, 3]. In recent years, the incidence has increased [4, 5]. AK cases related to contact lenses have also been reported in other countries [6, 7]. Although contact lens use is not the most common risk factor in China [8], AK continues to be seen in contact lens wearers, especially users of orthokeratology.

A good prognosis of AK depends on an early diagnosis, but early diagnosis remains a challenge. Several methods such as corneal scraping and confocal microscopy are fast and useful for diagnosis in the early stages. In some cases of early-stage AK, trophozoites were active, and the number of cysts was small. Diagnosis by in vivo confocal microscopy may be difficult, and direct examination of trophozoites by 0.9% sodium chloride (NaCl) may be helpful. In this retrospective study, the clinical and microbiological data of AK related to contact lens use were analysed in a tertiary hospital in Beijing, China.

## Methods

A retrospective review of the medical records of patients diagnosed with AK associated with contact lens use from January 2000 to December 2017 at Beijing Tongren Hospital was performed. This study was approved by the Institutional Review Board of Beijing Tongren Hospital and adhered to the tenets of the Declaration of Helsinki.

Cases were included in this study if corneal scraping and/or culture were positive for *Acanthamoeba*. The corneal samples were Giemsa stained for the detection of cysts. The material obtained in the next scraping sample was combined with 0.9% NaCl on a clean sterile glass slide as a wet mount to detect the trophozoites by direct microscopy (Olympus BX51, Tokyo, Japan); the cysts could also be detected. The images were captured using a colour digital camera (DP-12, Olympus). The samples were streaked onto non-nutrient agar plates covered with *Escherichia coli* and incubated at 28 °C for 5 to 15 days for the culture of amoebae. When corneal scraping and culture results were negative, cases were identified if *Acanthamoeba* cysts were observed by in vivo confocal microscopy (Heidelberg Engineering GmbH, Dossenheim, Germany) and typical clinical signs were present. The following data were collected: demographic information, lens type, history, risk factors, disease stages, and simultaneous bacteria and fungi culture reports. PCR was performed to determine the genotype of the *Acanthamoeba* isolates. The DNA extraction and PCR assays were performed as described in our previous study [9]. The PCR assay was performed with the genus-specific primers JDP1 (5′-GGCCCAGATCGTTTACCGTGAA) and JDP2 (5′- TCTCACAAGCTGCTAGGGGAGTCA). Then, direct sequencing of the PCR products was performed with the conserved primer 892C (5′-GTCAGAGGTGAAATTCTTGG).

Treatments were also analysed. The disease stages (Table 1) and treatments referred to our previous study [8]. Topical anti-amoebic therapy included chlorhexidine and/or polyhexamethylene biguanide (PHMB). The concentrations of these drugs were determined according to the disease stage, and oral itraconazole was administered to some patients. If the infectious process spread despite aggressive medical therapy or the case of AK was severe, therapeutic keratoplasty was performed. If the patients were coinfected with *Acanthamoeba* and other pathogens, appropriate treatment was administered simultaneously.

**Table 1:** The disease stages [8]

Stage	Manifestation
Early stage	Pseudodendritic or punctate corneal epithelial lesions, dot infiltrations under the epithelium, recurrent epithelial erosion, radial corneal neuritis, corneal ulcer smaller than 4 mm in diameter in the anterior stroma
Advanced stage	Deep stromal ulcer greater than 5 mm and less than 8 mm in diameter
Late stage	The deep stromal ulcer greater than 8 mm in diameter and hypopyon, central corneal thinning and perforation

Statistical analyses were performed using the SPSS statistical software package (SPSS for Windows, version 17.0; SPSS, Inc., Chicago, IL). Independent-sample t-tests and chi-square tests were conducted to compare the differences in the clinical data. A *P* value less than 0.05 was considered statistically significant.

## Results

**Fig. 1:**
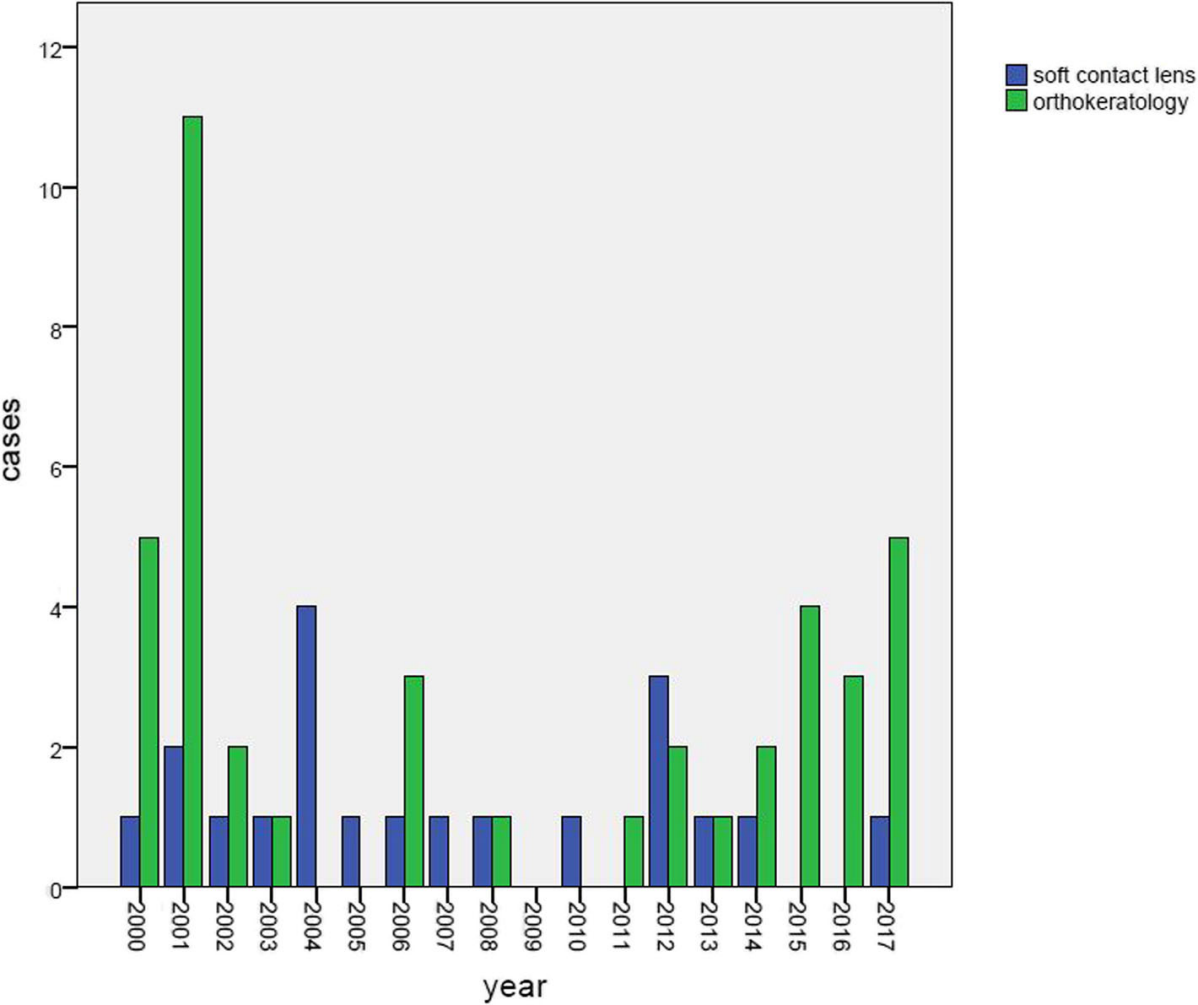
Cases of *Acanthamoeba* keratitis related to contact lens use in each year

There were 61 patients (64 eyes) included in the study; 24 (39.3%) patients were male and 37 (60.7%) patients were female. The age varied between 9 and 48 years old (mean 19.95 ± 7.06 years old). Of the 61 patients, 20 patients (32.8%) wore SCLs, and 41 (67.2%) patients used overnight orthokeratology. There were 3 bilateral cases in this study, and all of these patients used orthokeratology. The cases in each year are shown in Fig. 1 (Additional file 1); the incidence peaked in 2001.

**Fig. 2:**
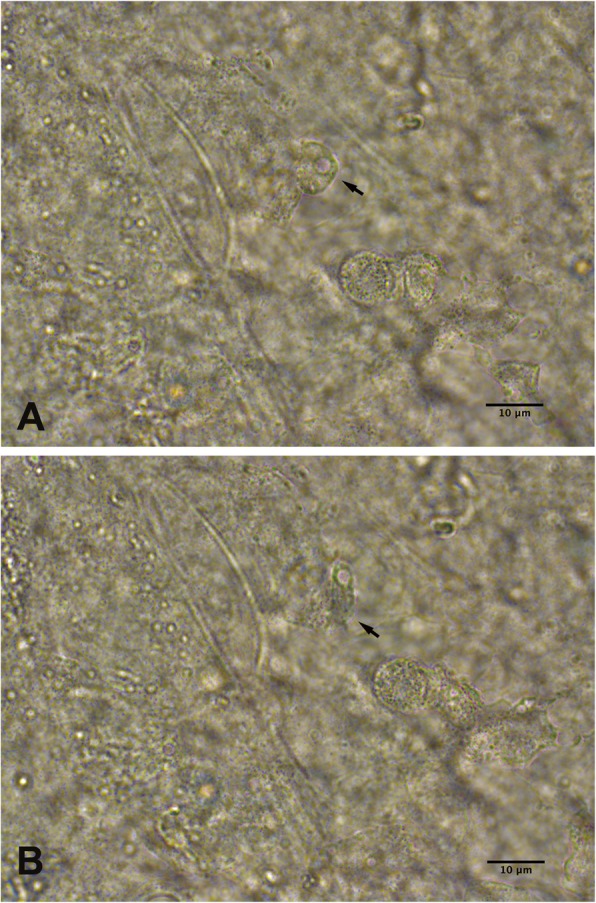
*Acanthamoeba* trophozoite in a 0.9% sodium chloride wet mount (black arrow) **a** The trophozoite was oval with a large nucleolus. **b** The same *Acanthamoeba* trophozoite became elongated in shape after several seconds

Table 2 shows the demographic characteristics, clinical features, microbiological culture results and treatments for all the cases. There was no significant difference in gender between the SCL and orthokeratology groups (chi-square test, *χ*^2^ = 0.005, *P* = 0.942). The orthokeratology wearers were younger than the patients who wore SCLs (independent-sample t-test, *t* = 4.338, *P* = 0.000). The symptom duration ranged from 5 days to 3 months. Fifty-four eyes (84.4%) had positive results according to the Giemsa-stained smears. In addition, trophozoites were detected in 19 eyes (29.7%) by 0.9% NaCl wet mounts. When the 0.9% NaCl wet mount was directly examined, the trophozoite had an oval or elongated outline, a large central nucleolus and hyaline pseudopodia (Fig. 2); it moved slowly with its pseudopodia in the tissue. In early-stage cases (20 eyes), 65% (13 eyes) had positive results according to the Giemsa-stained smears, and trophozoites were seen in 7 eyes (35%) on the 0.9% NaCl wet mounts. Six eyes (30%) were diagnosed by confocal microscopy combined with clinical signs. The culture-positive rate of *Acanthamoeba* was 76.6% (49/64). Six cases (9.84%) had polymicrobial infection, and most (5/6) were soft-contact lens wearers. The isolated organisms were *Pseudomonas aeruginosa* and *Staphylococcus epidermidis.* No fungal organisms were isolated. The genotypes of 9 isolates from nine patients were identified. Four patients were male, and 5 patients were female. Six isolates were from soft-contact lens wearers, and 3 isolates were from patients who used orthokeratology lenses. All of the *Acanthamoeba* strains belonged to genotype T4. Among the 9 strains, two were identified as T4/6, and the others were identified as T4/8, T4/9, T4/13, T4/24, T4/31, T4/34, and T4/41, respectively.

**Table 2:** Results of demographic characteristic, clinical feature, microbiological culture and the treatment in each lens type

Characteristic	Soft contact lens (*n* = 20,,eyes = 20)	Orthokeratology (*n* = 41,eyes = 44)
Gender, n(%)		
Female	12 (60.0)	25 (61.0)
male	8 (40.0)	16 (39.0)
Age (years)	25.90 ± 8.81	17.05 ± 3.41
Positive result in culture for *Acanthamoeba*, eyes(%)	16 (80.0)	33 (75.0)
Positive result in culture for bacteria, eyes(%)	5 (25.0)	1 (2.3)
*Pseudomonas aeruginosa*	3 (15)	0 (0)
*Staphylococcus epidermidis*	2 (10)	1 (2.3)
Treatment, eyes (%)		
Medical therapy	16 (80.0)	33 (75.0)
Therapeutic penetrating keratoplasty	4 (20.0)	11 (25.0)

The different disease stages according to each lens type are summarized in Table 3. A total of 76.6% of the eyes were cured of corneal scars by medical therapy, and the remainder of the eyes underwent therapeutic keratoplasty. There was no recurrence after surgery. In the orthokeratology group, the number of patients who required therapeutic penetrating keratoplasty after 2005 (3/22) was less than that before 2005 (8/19) (chi-square test, *χ*^2^ = 4.209, *P* = 0.04).

**Table 3:** The cases in each lens type with different disease stages

Lens type	Early stage, eyes(%)	Advanced stage, eyes(%)	Late stage, eyes (%)
Soft contact lens	6 (30.0)	12 (60.0)	2 (10.0)
Orthokeratology	14 (31.8)	20 (45.5)	10 (22.7)

In the orthokeratology group, 87.8% (36/41) of the patients rinsed their lenses and/or cases with tap water, and in the SCL group, 11 patients (55%) wore their lenses while showering (5 cases, 25.0%) or sleeping (6 cases, 30.0%).

## Discussion

The *Acanthamoeba* genus is a free-living amoebae that can be found in the air, soil and water. In China, the most common risk factor is ocular trauma, followed by contact lens wear [8]; however, cases related to contact lens use were seen almost yearly in this study. In addition, a notable finding of our previous study was that the number of cases of AK was nearly one-half the number of cases of microbial keratitis related to contact lens wear [10]. However, few studies have reported the clinical and microbiological features of contact lens-related AK in China.

In this study, the peak year of occurrence was 2001, which may be due to the outbreak of AK associated with orthokeratology in 2001 [11, 12]. At that time, the government intervened to regulate the orthokeratology market, and the number of patients fitted with orthokeratology decreased. The cases of AK related to orthokeratology decreased correspondingly [13]. In this study, there were no patients with cases related to orthokeratology in 2004 and 2005. However, in recent years, cases have reemerged and increased slightly. This trend has also been seen in other countries. It was reported that the new baseline incidence of AK in the United States is 10 times greater than that before 2004 [14]. A study of AK among rigid gas-permeable contact lens wearers showed that nearly a quarter of patients were orthokeratology lens wearers from 2005 to 2011 [15]. In New Zealand, the incidence of AK in 2009 to 2016 had more than doubled when compared with the preceding 7-year period [4].

In previous studies, most cases related to contact lenses involved soft lenses [4, 6, 16, 17]. However, more than two-thirds of AK cases were related to orthokeratology in this study. Even in Hong Kong, 37.5% of contact lens-related AK was attributed to orthokeratology [18]. The reason may be that the myopic population in China has increased in recent years, and there has also been an increase in the average degree of myopia, especially among young children [19]. Orthokeratology has become one of the most popular corrective choices for parents. Consequently, many accompanying adverse effects associated with orthokeratology, such as AK, have been observed. Moreover, most orthokeratology users are children and teenagers; consequently, and the orthokeratology-associated AK patients were younger than the patients who used soft lenses. Therefore, it is necessary that patients and their parents learn about proper lens hygiene and care. Unlike other studies [4], the bilateral cases in this study were all associated with orthokeratology.

Early diagnosis is essential to ensure a good prognosis. Some studies have suggested that it is important to remember and consider AK in all cases of contact lens keratitis [20, 21]. Clinical features of AK are similar to other types of keratitis, such as fungal and herpes simplex virus infections. Although confocal microscopy is a noninvasive technique [1], the images of the cysts were atypical in some of the early-stage cases [22]. The microbiological examination of corneal scrapings by microscopy is easy, fast and efficient. In this study, the positive rate of Giemsa-stained smears reached 84.4%. In the early-stage cases, the rate was relatively low, while the positive rate of the 0.9% NaCl wet mounts for the detection of trophozoites was slightly increased. Because drugs were not administered in some of the early-stage cases, encystment did not occur. Trophozoite activity can be observed directly by 0.9% NaCl wet mounts. For highly suspected early-stage cases, especially those characterized by epitheliopathy, examinations with Giemsa-stained smears, 0.9% NaCl wet mounts and confocal microscopy should be performed to help the early diagnosis of AK.

Culturing for *Acanthamoeba* was required for any patient suspected of having infective keratitis. The culture-positive rate of *Acanthamoeba* has been reported to be between 30 and 60% [20]. Even though culture requires a long incubation time, it is still the definitive diagnosis method [23]. In this study, the positive culture rate (76.6%) was higher than that in previous reports, possibly because two-thirds of the cases were diagnosed in the advanced and late stages, and the corneal scrapings may have included more trophozoites and cysts.

It is notable that AK can cause coinfection with other organisms. It was reported that coinfections with bacteria were seen in 23% of cases [24]. In a study in Egypt, bacteria were isolated from all the patients with AK [7]. In this study, 9.84% of the patients presented polymicrobial infection. This was similar to the result of our previous study (15%) [25]. Because this survey was conducted in a tertiary referral centre, many cases received antibacterial treatment before anti-amoebic therapy. The result of the genotyping analysis was consistent with our previous study [9] and other studies in China [26]. Although the subtypes differ, genotype T4 is still the main AK-related genotype in China; furthermore, genotype T4 is the predominant genotype in other countries and areas [27–30].

To date, no drugs have been specifically approved for AK by the Food and Drug Administration [23]. Biguanides, including chlorhexidine and PHMB, are commonly used. In this study, more than two-thirds of the eyes were cured with medical therapy. Therapeutic penetrating keratoplasty is required for severe cases of AK, but it is not recommended as a method for removal of the organisms from the cornea [20] because it is associated with poor outcomes, such as poor graft survival, repeat transplantation and glaucoma. Postoperative topical anti-amoeba drug therapy was also required for the patients in this study to avoid recurrence. In this study, the rate of therapeutic keratoplasty decreased after 2005 compared with the rate before 2005 in the orthokeratology group. There are several possible reasons. First, since 2003, orthokeratology has gradually entered into a state of relatively standard and healthy development in China [31]; additionally, clinicians gradually became highly suspicious of AK in keratitis related to contact lens use. Contact lens users were told to go to a hospital quickly if they had symptoms. Second, as confocal microscopy has high sensitivity and specificity in the diagnosis of AK, some cases were diagnosed rapidly. Third, as the number of cases increased, clinicians became more experienced in treatment.

The risk behaviours of orthokeratology lens and soft lens wearers were slightly different in this study. Approximately 87.8% of patients who used orthokeratology lenses rinsed their lenses with tap water, while the most common risk behaviours in SCL wearers were showering and sleeping while wearing their lenses. The risk behaviours were consistent with a survey in Egypt [7]. However, in the United States, storing lenses in tap water and topping off contact lens solution were the most common risk factors for AK in rigid gas-permeable contact lens wearers. Moreover, wearing lenses for orthokeratology overnight was also thought to be a risk factor [15]. Contact lens wearers, especially orthokeratology patients, should strictly adhere to good contact lens hygiene practices and avoid lens exposure to tap water to minimize the risk of AK.

There were some limitations to this study. As a retrospective study, some information, such as history, was incomplete during clinical data collection. Because this survey was conducted in one hospital, there might be bias in the percentage of cases associated with each lens type. More work is needed to explain the difference in the polymicrobial infection rate between orthokeratology lens and SCL wearers.

## Conclusions

In conclusion, AK is one of the most challenging infections to manage. Although contact lens wear is not the most common risk factor for AK in China, AK cases related to contact lenses have been observed continuously over the years. More than two-thirds of cases were associated with orthokeratology lenses. The most common risk behaviour of orthokeratology users was rinsing lenses and/or cases with tap water, while in SCL users, it was wearing lenses while showering or sleeping. For highly suspected early-stage cases of, especially those characterized by epitheliopathy, examinations with Giemsa-stained smears, 0.9% NaCl wet mounts and confocal microscopy should be performed to help the early diagnosis of AK. With increasing awareness of AK by clinicians, early diagnosis and effective medical treatment, the rate of therapeutic keratoplasty has decreased in recent years in orthokeratology users. Optometrists and ophthalmologists should teach contact lens wearers, especially orthokeratology users, to strictly adhere to good contact lens hygiene practices and avoid lens exposure to tap water to minimize the risk of AK.

## Additional file

Additional file 1: Attachment 1 Cases of Acanthamoeba keratitis related to contact lens in each year (DOCX 13 kb)

## Data Availability

The data used to support the findings of this study are available from the corresponding author upon request.

## Abbreviations

AK: *Acanthamoeba* keratitis
NaCl: Sodium chloride
SCL: Soft contact lens
