# Supplementary material for: Acanthamoeba keratitis related to contact lens use in a tertiary hospital in China
Source: BMC Ophthalmol. 2019 Sep 18;19:202. doi: 10.1186/s12886-019-1210-2 (PMC6751601; doi:10.1186/s12886-019-1210-2)
Supplement: Supplementary file 1 — Attachment 1 Cases of Acanthamoeba keratitis related to contact lens in each year (DOCX 13 kb) [file 12886_2019_1210_MOESM1_ESM.docx]

**Attachment 1** Cases of Acanthamoeba keratitis related to contact lens in each year

| Year | Soft contact lens | Orthokeratology |
| --- | --- | --- |
| 2000 | 1 | 5 |
| 2001 | 2 | 11 |
| 2002 | 1 | 2 |
| 2003 | 1 | 1 |
| 2004 | 4 | 0 |
| 2005 | 1 | 0 |
| 2006 | 1 | 3 |
| 2007 | 1 | 0 |
| 2008 | 1 | 1 |
| 2009 | 0 | 0 |
| 2010 | 1 | 0 |
| 2011 | 0 | 1 |
| 2012 | 3 | 2 |
| 2013 | 1 | 1 |
| 2014 | 1 | 2 |
| 2015 | 0 | 4 |
| 2016 | 0 | 3 |
| 2017 | 1 | 5 |
